# Intrinsic Fluorescence of PAMAM Dendrimers—Quenching Studies

**DOI:** 10.3390/polym10050540

**Published:** 2018-05-17

**Authors:** Malgorzata Konopka, Anna Janaszewska, Barbara Klajnert-Maculewicz

**Affiliations:** Department of General Biophysics, Faculty of Biology and Environmental Protection, University of Lodz, 141/143 Pomorska St., 90-236 Lodz, Poland; malgorzata.konopka@biol.uni.lodz.pl (M.K.); ankuj@poczta.onet.pl (A.J.)

**Keywords:** PAMAM, intrinsic emission, autofluorescence, quenching

## Abstract

Intrinsic, non-traditional fluorescence of polyamidoamine (PAMAM) dendrimers that do not possess classical fluorophores has been attracting considerable interest for the last decade. Many hypotheses regarding the source of the fluorescence have appeared, but some of them are still disputable. In order to shed new light on the nature of the phenomenon, we applied quenchers that are normally used to study intrinsic fluorescence of proteins (i.e., KI, CsCl, and acrylamide). KI and acrylamide efficiently quenched steady state fluorescence of PAMAM G2, PAMAM G3, and PAMAM G4 dendrimers. Stern-Volmer plots exhibited a downward curvature that has been elucidated by heterogenous emission. We assume that there are two distinct fluorescent moieties in the dendrimer structure that are characterized by different accessibility to the quenchers.

## 1. Introduction

Polyamidoamine (PAMAM) dendrimers belong to dendritic polymers that have been the most intensively studied. These regular, monodisperse, hyperbranched macromolecules—possessing a core molecule, layers of branched monomers, and plenty of terminal groups—attract interest due to their many potential applications, especially in a biomedical field [1,2]. Researchers frequently used different fluorescent probes to investigate properties of dendrimers. Surprisingly, they observed a weak fluorescent background in the blue region that was firstly believed to be due to contamination, but when the observations were continuously repeated, the effect was finally attributed to PAMAM dendrimers, and believed to be their intrinsic fluorescence [3,4,5]. The fluorophore has remained a subject of a debate, because PAMAM dendrimers do not possess conventional chromophores. In 2001, Larson and Tucker published the first article fully devoted to the intrinsic fluorescence of carboxylate-terminated PAMAM dendrimers, where they attributed the fluorescence to an n→π* transition from amido groups throughout the dendritic structure [6]. Later reports concentrated on the presence of tertiary amines [7,8]. The last three years have witnessed a substantial growth in the number of publications devoted to non-traditional intrinsic fluorescence of dendrimers and other polymers. There is no doubt that the exciting [non-traditional intrinsic fluorescence NTIF] phenomenon requires a significant paradigm shift from traditional thinking about fluorescence as it was described in the review by Yuan and Zhang [9].

It has been found that fluorescence emission of PAMAM dendrimers is a consequence of the existence of imidic acid, imine, and tertiary ammonium groups in their structure [10]. Although it is believed that the presence of tertiary amines is of crucial importance, the nature of the intriguing phenomenon of intrinsic fluorescence has remained controversial, and a successful explanation has not been achieved to fully elucidate their mechanism.

In order to shed light on the subject, we have employed a steady-state fluorescence quenching method using three classical quenchers: acrylamide, cesium chloride, and potassium iodide. The quenchers differ in terms of the size and the charge, and therefore, they have a different ability to diffuse to the fluorophores throughout the dendritic structure. To be able to draw conclusions on the role of the internal structure of dendrimers on the quenching process, three generations of PAMAM dendrimers have been studied (G2, G3, and G4). Low generations of dendrimers have an open structure, whereas higher generations adopt a three-dimension architecture with densely packed surface terminal groups.

## 2. Materials and Methods

### 2.1. Materials

Polyamidoamine (PAMAM) dendrimers generations 2, 3, and 4 (G2, G3, and G4), acrylamide, cesium chloride, and potassium iodide were obtained from Sigma-Aldrich (St. Louis, MO, USA). All other chemicals were of analytical grade. Water used to prepare solutions was double-distilled.

### 2.2. Methods

The dendrimers were dissolved in phosphate-buffered saline (PBS: 150 mmol/L NaCl, 1.9 mmol/L NaH_2_PO_4_, 8.1 mmol/L Na_2_HPO_4_, pH 7.4) at a concentration of 1 mmol/L. Excitation and emission spectra were taken with a Perkin-Elmer LS-55 spectrofluorometer (Lodz, Poland) at 24 °C. Excitation and emission slit widths were set to 7 and 5 nm, respectively. The maxima of the excitation wavelength were slightly dependent on the dendrimer generation, and for G2, G3, and G4, equaled to 333 nm, 330 nm, and 334 nm, respectively. Dendrimers were excited by the wavelength at the maximum of excitation. The emission spectra were recorded from 350 to 570 nm. We checked that dendrimers showed no absorbance neither for λ_exc_ nor λ_em_ (Figure S1 in Supplementary Materials).

Fluorescence quenching studies were carried out with acrylamide, potassium iodide, and cesium chloride. The stock solutions for acrylamide, KI, and CsCl were 8 mol/L, 10 mol/L, and 5 mol/L, respectively. A stock solution of KI contained 0.1 mmol/L Na_2_S_2_O_3_ to prevent oxidation of I^−^ to I_3_^−^. Increasing aliquots of the quencher were added from a stock solution to a 1 cm path length quartz cuvette with 1 mmol/L dendrimer. The sample in the cuvette was continuously stirred. The emission spectra were recorded. It was checked that dilution of the sample did not affect the intensity of the emission due to the fact that small aliquots of the quenchers were added to the cuvette.

## 3. Results

**Figure 1:**
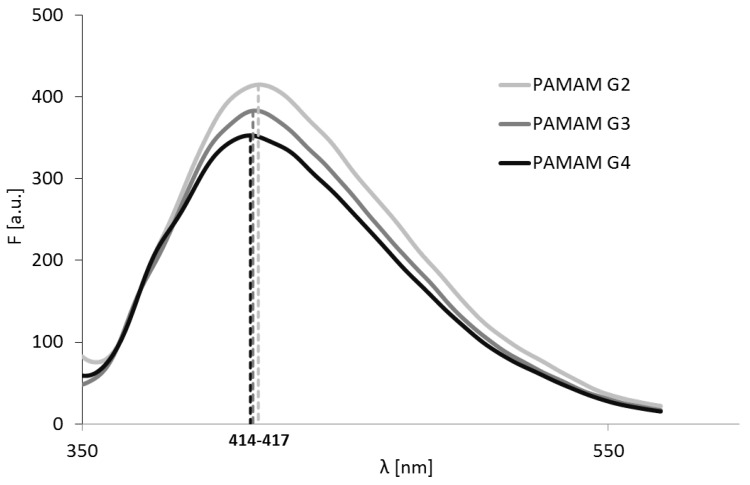
Emission spectra of PAMAM dendrimers (c = 1 mmol/L). λ_exc_ = 333 nm, 330 nm, and 334 nm for PAMAM G2, PAMAM G3, and PAMAM G4, respectively.

PAMAM dendrimers exhibited intrinsic fluorescence. The position of the maximum of emission intensity depended on the dendrimer generation (Table 1). The strongest fluorescence was observed for PAMAM G2, and the weakest for PAMAM G4 (Figure 1). Maximal fluorescence intensity was in inverse proportion to the dendrimer generation. This result is consistent with previous observations made at the similar pH [11]. For all generations, the spectra were not symmetrical, and deconvolution of the spectra revealed that there were two spectrofluorimetric components that contributed to the emission, with the maximum wavelength at approximately 410 nm and 455 nm (Figure S2 in Supplementary Materials). Additionally, at approximately 373 nm, we observed a Raman band.

Fluorescence quenching experiments were carried out using three different quenchers: neutral acrylamide, cationic CsCl (Cs^+^), and anionic KI (I^–^). In the case of CsCl, we observed no quenching effect. On the contrary, acrylamide and KI quenched the fluorescence (Figures S3–S8 in Supplementary Materials). A more pronounced effect was observed for KI.

**Figure 2:**
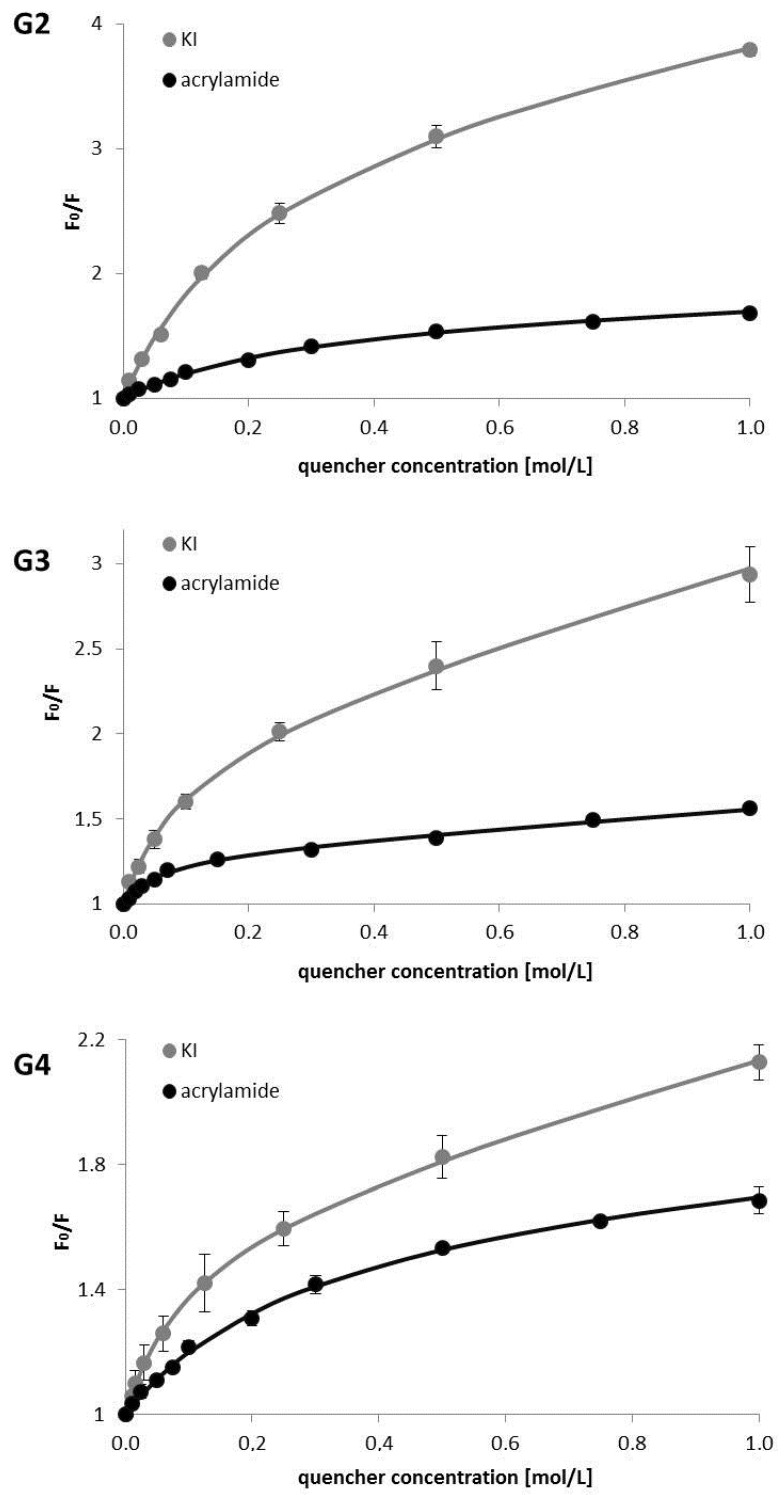
Stern-Volmer plots for PAMAM G2, PAMAM G3, and PAMAM G4 quenched by acrylamide and KI, *n* = 3.

The correlations between the concentration of the quencher and the decrease in fluorescence intensity were presented by means of Stern-Volmer plots (Figure 2). In order to determine Stern-Volmer plots, the fluorescence intensity was measured for λ_max em_ of each PAMAM dendrimer.

For all studied systems, the Stern-Volmer plots curved downwards towards the X-axis, which is characteristic for fractional accessibility of fluorimetric sites to quenchers. The Stern-Volmer parameters were estimated from the obtained curves using non-linear least-squares analysis based on the equations

(1) $$\frac{F}{F_{0}}=\overset{2}{\underset{i=1}{\sum }}\frac{f_{i}}{1+K_{{\mathrm{SV}}_{i}}\left[Q\right]}$$

(2) $$\sum_{i=1}^{2}f_{i}=1$$

where

F_0_—fluorescence intensity in the absence of the quencher,

F—fluorescence intensity in the presence of the quencher at a concentration [Q],

K_SV_—Stern-Volmer constant,

f_i_—fractional intensity of a component i.

The results are summarized in Table 2.

## 4. Discussion

Fluorescence experiments provide information on the molecular environment of a chromophore. This is especially important in the case of intrinsic fluorescence of systems that do not possess classical fluorophores, and for which the source and the mechanism of the photoluminescence have not yet been understood completely. To make progress in deciphering the puzzle of intrinsic fluorescence of PAMAM dendrimers, we employed three quenchers: acrylamide, KI, and CsCl, that are often used in studying proteins. Dendrimers resemble proteins in terms of size and shape, therefore, they are sometimes described as artificial proteins [12]. The mimicry of proteins has led to numerous applications of dendrimers in biomedical fields, such as gene therapy, drug delivery, enzyme-like catalysis, light harvesting, and many others.

Fluorescence quenching occurs via physical contact between the quencher and the fluorophore, and hence, it is directly dependent on the extent to which they can approach to each other. Quenching by ionic quenchers happens due to a heavy ion effect that requires a direct collision of the ions with the excited fluorophore. In the case of Cs^+^, there was no such collision, probably because of the presence of cationic charge on the surface of PAMAM dendrimers that possess primary amine terminal groups. At neutral pH, the primary amines on the surface of PAMAM dendrimers are protonated [13], and they might be responsible for repulsion of Cs^+^.

**Figure 3:**
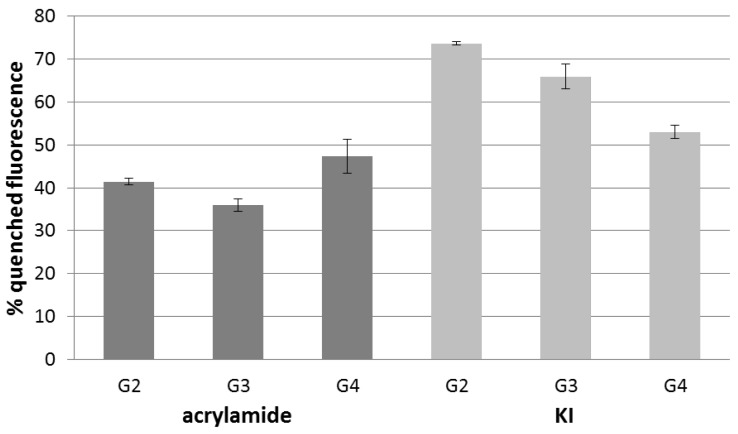
Percent of quenched fluorescence of PAMAM G2, PAMAM G3, and PAMAM G4 using 1 mmol/L acrylamide (dark grey bars) and 1 mmol/L KI (light grey bars), *n* = 3.

On the contrary, KI turned out to be an efficient quencher. We assume that collision of I^–^ with the excited fluorophore is driven by an electrostatic interaction between anionic iodium ions and cationic dendrimer groups. It seems that I^–^ can penetrate the interior of the dendrimer to some extent. This ability decreases with an increasing generation of the dendrimer. It explains why the percent of quenched fluorescence was the biggest for G2 (74%), and the smallest for G4 (53%) (Figure 3). The observed linear decrease in quenching corresponds with changes in the dendrimer architecture. The dendrimer has an open structure for lower generations, and becomes densely packed for higher generations. An open structure of lower generations provides an easy access route to fluorophores, whereas higher generations create a hurdle for quenchers. A similar dependence was observed for quenching intrinsic fluorescence of triazine dendrimers by KI that was used to probe porosity of the dendrimers [14].

The last tested quencher was acrylamide, that is polar but uncharged. Acrylamide quenched the intrinsic dendrimer fluorescence and the process was not dependent on the generation. Quenching the tryptophan fluorescence by acrylamide is widely applied in biophysical studies of protein structures. Acrylamide is able to quench tryptophan residues that are fully buried in the globular fold and separated from the solvent [15,16,17,18]. It happens because acrylamide is able to penetrate into the interior of the protein through diffusive processes enabled by small fluctuations in the protein conformation. This ability explains well why acrylamide quenched fluorophores in dendrimers of all generations to a similar extent.

There are very limited studies on quenching intrinsic fluorescence of dendrimers and other polymeric systems. Fluorescence of poly(propyl ether imine) dendritic macromolecules was quenched in the presence of aromatic compounds: nitrobenzene, 1,3-dinitrobenzene, and 1,3,5-trinitrobenzene [19]. Significant quenching of steady state fluorescence of PAMAM G3 dendrimers was observed upon addition of 2,4 dinitrotoluene [20]. In the above examples, Stern-Volmer plots exhibited an upward curvature that was elucidated by formation of a polymer–quencher complex. Therefore, fluorescence quenching was controlled by both collisional (dynamic) quenching, as well as static quenching caused by host–guest complex formation. Interestingly, in our experiments, all Stern-Volmer plots were curved downwards. This suggests heterogeneous emission that is a common case when there are two or more fluorescing centers. The best fit was achieved for two fluorescent components, which indicates that there are two kinds of fluorescence emitting moieties in the dendrimer structure. In the first paper that was fully devoted to the phenomenon of intrinsic fluorescence of dendrimers, Larson and Tucker (2001) recovered three distinct lifetimes of carboxylate-terminated PAMAM dendrimers (G2.5–G7.5). The shortest lifetime was attributed to background emission from the solvent, and the longer to the dendrimers. The authors concluded that either there was a single fluorescent moiety in two distinct structural environments, or two different fluorescent centers. Later, two discrete fluorescent lifetimes with similar values, as observed by Larson and Tucker, were found for amino-terminated PAMAM G4 dendrimers [10], and for hyperbranched PAMAM polymers [21], confirming inhomogeneity of the systems. So far, the most sophisticated studies on the nature of NTIF of PAMAM dendrimers were performed using quantum chemical theory methods [22]. It has been demonstrated that both imidic acid and tertiary ammonium can give fluorescence emission. These components are products of transformation of amide groups and tertiary amines, respectively. Tertiary amines are deeply buried in the core of the dendrimer, whereas amide groups are located at each layer of the dendrimer structure. It means that part of them are located deeper in the interior, and part closer to the surface. We have demonstrated that one of the moieties is significantly more strongly quenched, and K_SV_ has achieved approximately from 40 to 170 times higher values for this moiety. We assume that the large K_SV_ values might indicate that there is strong quenching of the chromophores that are located close to the surface of the dendrimer, and thus, that they are more mobile by being in the vicinity to the terminal groups. On the other hand, the small K_SV_ values could represent quenching of the chromophores that are located deeper in the interior of the dendrimer in a more rigid environment, and thus, they are less easily quenched. Accessibility to the quenchers has differed for these two fluorescent centers, and in the case of KI, it has depended on the dendrimer generation. It confirms the previous assumptions that one fluorescent moiety (imidic acid) is located closer to the surface, whereas the other (tertiary ammonium) is buried deeper in the structure of the dendrimer.

To sum up, our work proved that using conventional quenchers that are frequently applied in studying proteins is a promising approach that can help to improve knowledge of the mysterious nature of intrinsic, non-traditional fluorescence of dendrimers.

We confirmed that there are two distinct fluorescent moieties in the PAMAM dendrimers that differ in terms of location. For higher generations of PAMAM dendrimers (that have more dense structure) quenchers accessibility to the interior decreases.

Undoubtedly, further experimental investigations are needed in this area.

## Figures and Tables

**Table 1:** Spectrofluorimetric parameters of intrinsic emission of polyamidoamine (PAMAM) dendrimers.

Generation	λ_max_ (nm)	Stokes Shift (nm)	F_max_ (a.u.)
G2	417	84	415
G3	415	85	383
G4	414	80	353

**Table 2:** Estimated Stern-Volmer parameters.

Quencher	Generation	K_1_ [M^−1^]	*f*_1_	K_2_ [M^−1^]	*f*_2_	*R* ^2^
Acrylamide	G2	0.23 ± 0.10	0.68 ± 0.07	7.41 ± 2.35	0.32 ± 0.06	0.992
	G3	0.21 ± 0.04	0.76 ± 0.02	22.06 ± 4.50	0.24 ± 0.02	0.994
	G4	0.07 ± 0.02	0.56 ± 0.02	5.91 ± 0.50	0.44 ± 0.02	0.998
KI	G2	0.25 ± 0.21	0.28 ± 0.04	16.13 ± 2.01	0.72 ± 0.04	0.997
	G3	0.50 ± 0.14	0.47 ± 0.04	20.75 ± 2.94	0.53 ± 0.04	0.997
	G4	0.33 ± 0.04	0.59 ± 0.02	16.03 ± 1.24	0.41 ± 0.02	0.999

## Supplementary Materials

The following are available online at http://www.mdpi.com/2073-4360/10/5/540/s1, Figure S1: Absorbance spectra of PAMAM G2 (A), PAMAM G3 (B) and PAMAM G4 (C) dendrimers at a concentration of 1mmol/L in the absence of the quenchers and in the presence of the quenchers at a concentration of 0.2 mol/L,, Figure S2: Deconvolution of spectra of PAMAM G2 (A), PAMAM G3 (B) and PAMAM G4 (C) dendrimers, Figure S3: Fluorescence quenching of PAMAM dendrimer G4 (c = 1mmol/L) by KI, Figure S4: Fluorescence quenching of PAMAM dendrimer G4 (c = 1mmol/L) by acrylamide, Figure S5: Fluorescence quenching of PAMAM dendrimer G3 (c = 1mmol/L) by KI, Figure S6: Fluorescence quenching of PAMAM dendrimer G3 (c = 1mmol/L) by acrylamide, Figure S7: Fluorescence quenching of PAMAM dendrimer G2 (c = 1mmol/L) by KI, Figure S8: Fluorescence quenching of PAMAM dendrimer G2 (c = 1mmol/L) by acrylamide.
