# Supplementary material for: Intrinsic Fluorescence of PAMAM Dendrimers—Quenching Studies
Source: Polymers (Basel). 2018 May 17;10(5):540. doi: 10.3390/polym10050540 (PMC6415388; doi:10.3390/polym10050540)
Supplement: Supplementary file 1 [file polymers-10-00540-s001.pdf]

polymers

MDPI

Intrinsic fluorescence of PAMAM dendrimers – quenching studies

Malgorzata Konopka [1], Anna Janaszewska [1] and Barbara Klajnert-Maculewicz [1,*]

[1] Department of General Biophysics, Faculty of Biology and Environmental Protection, University of Lodz, 141/143 Pomorska St., 90–236 Lodz, Poland; malgorzata.konopka@biol.uni.lodz.pl (M.K.); ankuj@poczta.onet.pl (A.J.)

* Correspondence: barbara.klajnert@biol.uni.lodz.pl.; Tel.: +48 42 635 44 29

Supporting Information

Introduction

Polyamidoamine (PAMAM) dendrimers generations 2, 3, and 4 (G2, G3, and G4), acrylamide, cesium chloride, and potassium iodide were obtained from Sigma-Aldrich. All other chemicals were of analytical grade. Water used to prepare solutions was double-distilled. The dendrimers were dissolved in phosphate-buffered saline (PBS: 150 mmol/L NaCl, 1.9 mmol/L NaH2PO4, 8.1 mmol/L Na_2HPO_4, pH 7.4) at a concentration of 1 mmol/L. Excitation and emission spectra were taken with a Perkin-Elmer LS-55 spectrofluorimeter at 24 °C. Excitation and emission slit widths were set to 7 and 5 nm, respectively. The maxima of the excitation wavelength were slightly dependent on the dendrimer generation and forG2, G3, and G4 equaled to 333 nm, 330 nm, and 334 nm, respectively. Dendrimers were excited by the wavelength at the maximum of excitation. The emission spectra were recorded from 350 to 585 nm. Fluorescence quenching studies were carried out with acrylamide, potassium iodide, and cesium chloride. The stock solutions for acrylamide, KI, and CsCl were 8 mol/L, 10 mol/L, and 5 mol/L, respectively. A stock solution of KI contained 0.1 mmol/L $Na_2S_2O_3$ to prevent oxidation of I^- to I^{3-}. Increasing aliquots of the quencher were added from a stock solution to a 1-cm path length quartz cuvette with 1 mmol/L dendrimer. The sample in the cuvette was continuously stirred. The emission spectra were recorded.

A

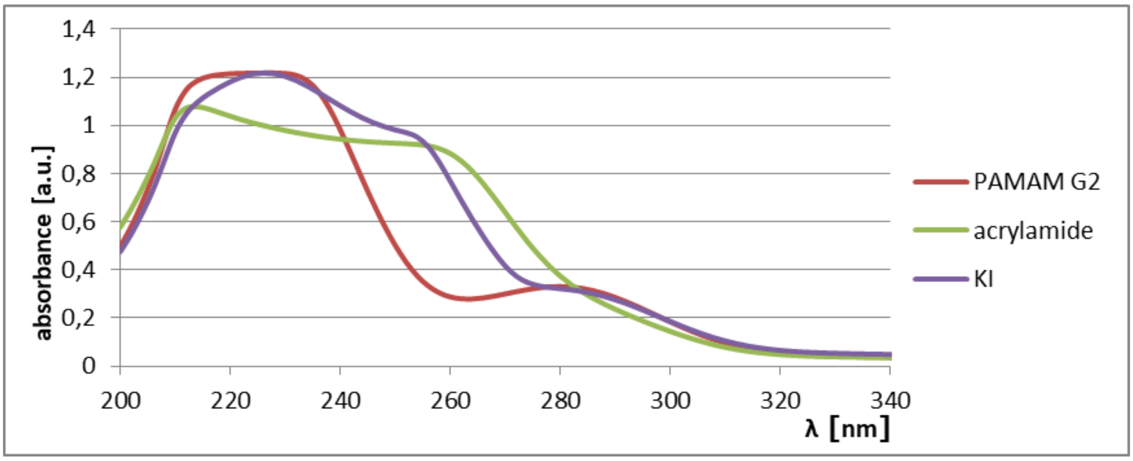

B

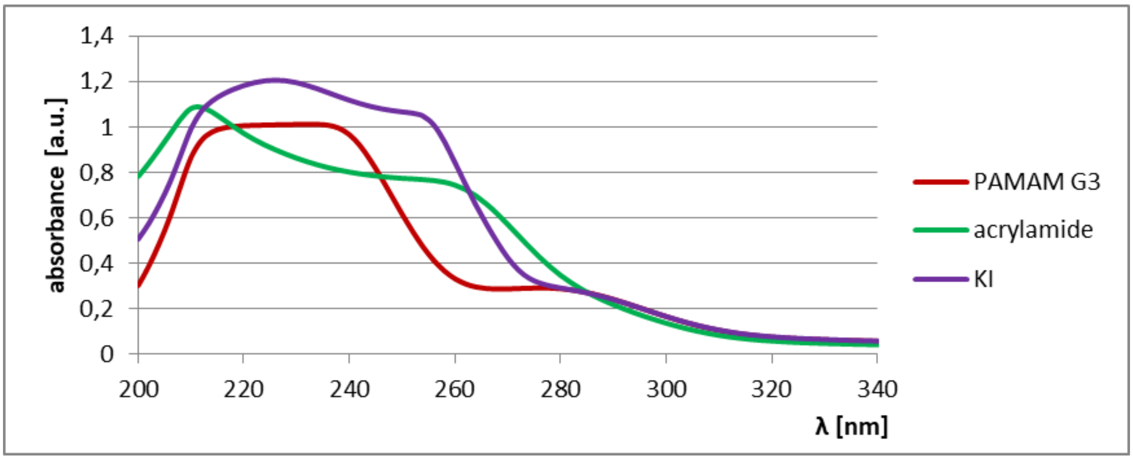

C

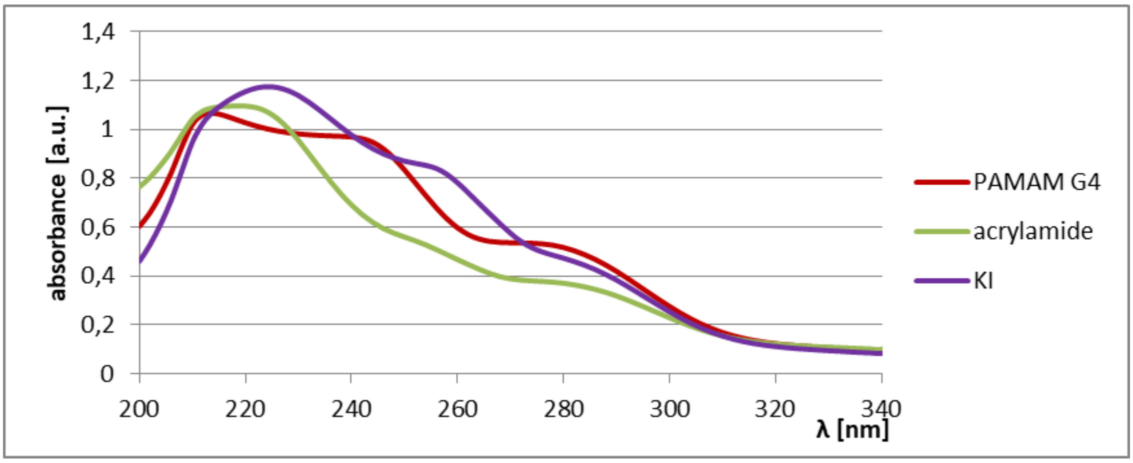

Fig. S1. Absorbance spectra of PAMAM G2 (A), PAMAM G3 (B) and PAMAM G4 (C) dendrimers at a concentration of 1mmol/L in the absence of the quenchers and in the presence of the quenchers at a concentration of 0.2 mol/L.

A

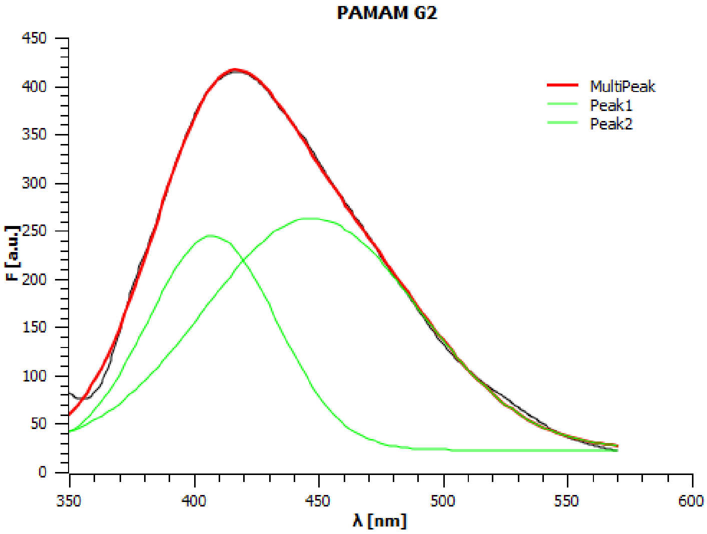

B

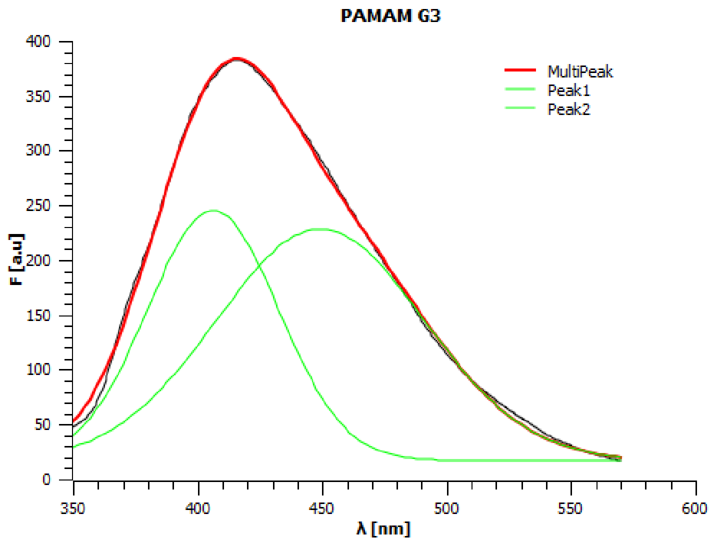

C

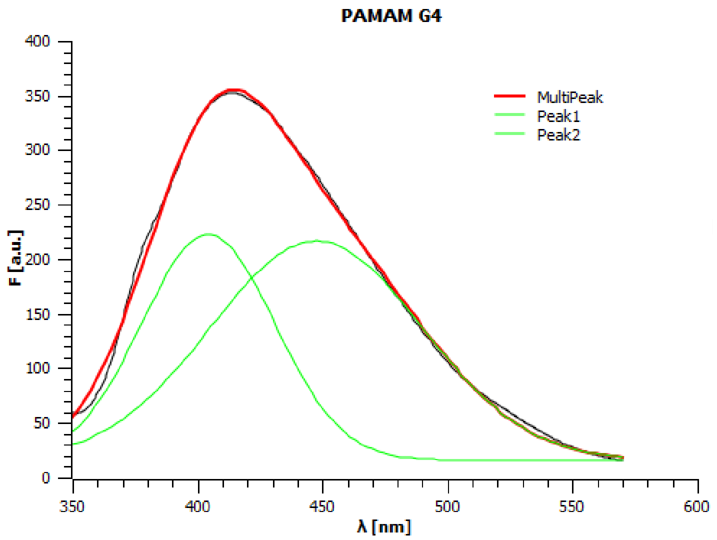

Fig. S2. Deconvolution of spectra of PAMAM G2 (A), PAMAM G3 (B) and PAMAM G4 (C) dendrimers.

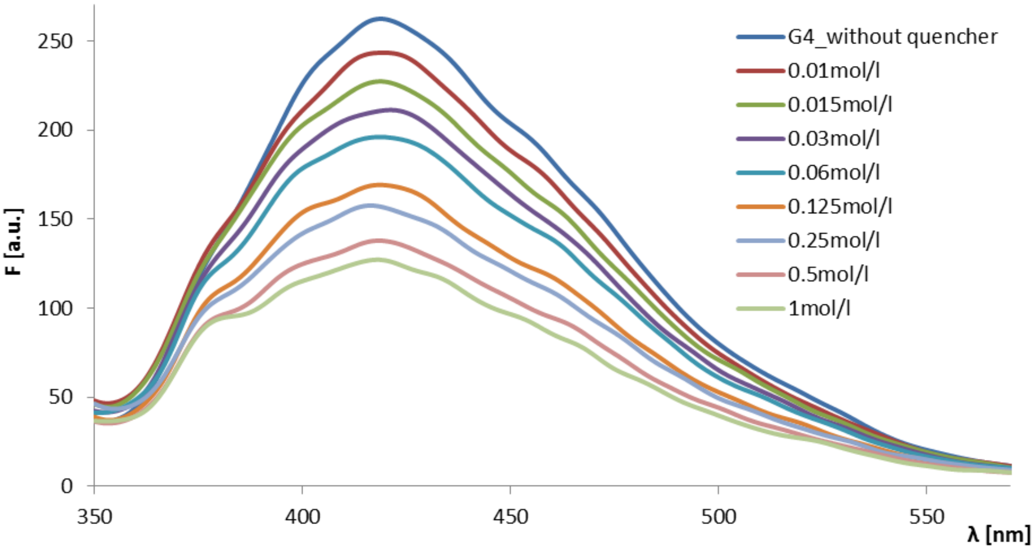

Fig. S3. Fluorescence quenching of PAMAM dendrimer G4 (c=1mmol/L) by KI.

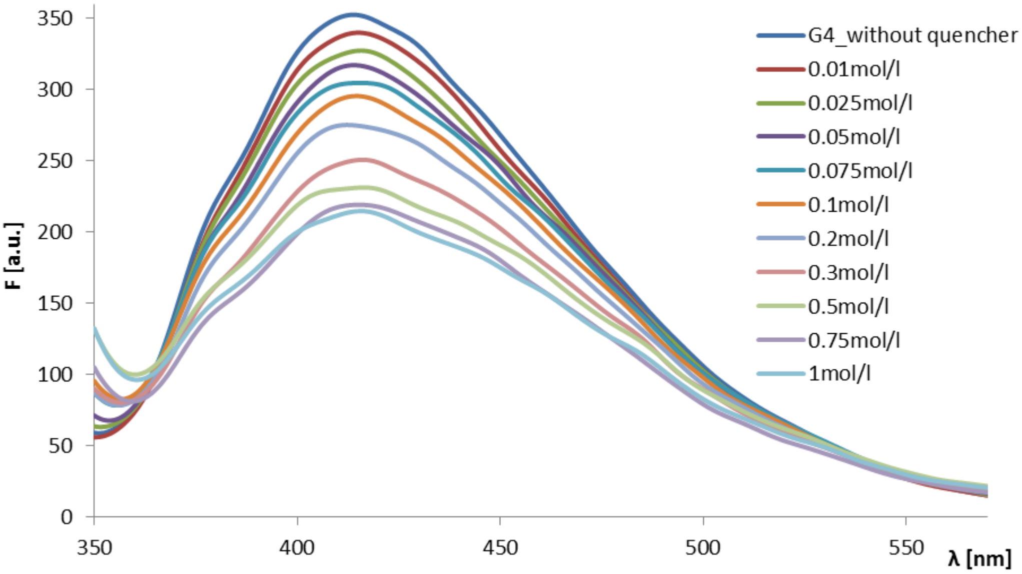

Fig. S4. Fluorescence quenching of PAMAM dendrimer G4 (c=1mmol/L) by acrylamide.

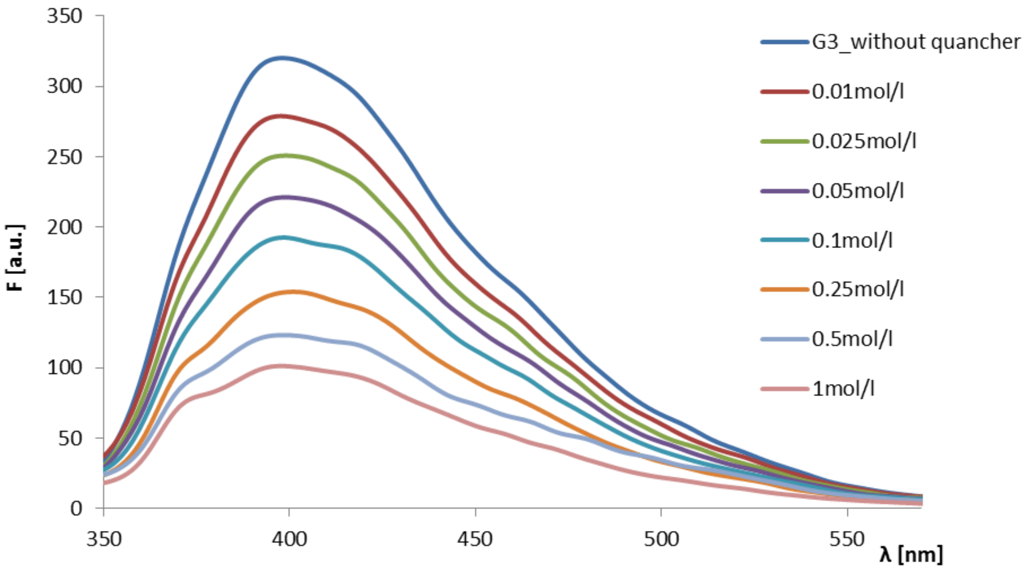

Fig. S5. Fluorescence quenching of PAMAM dendrimer G3 (c=1mmol/L) by KI.

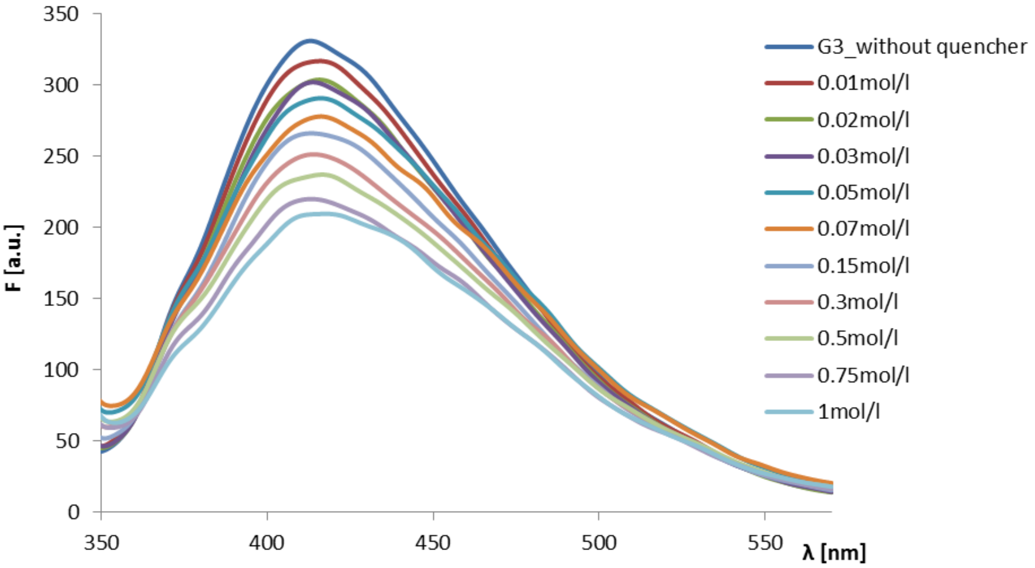

Fig. S6. Fluorescence quenching of PAMAM dendrimer G3 (c=1mmol/L) by acrylamide.

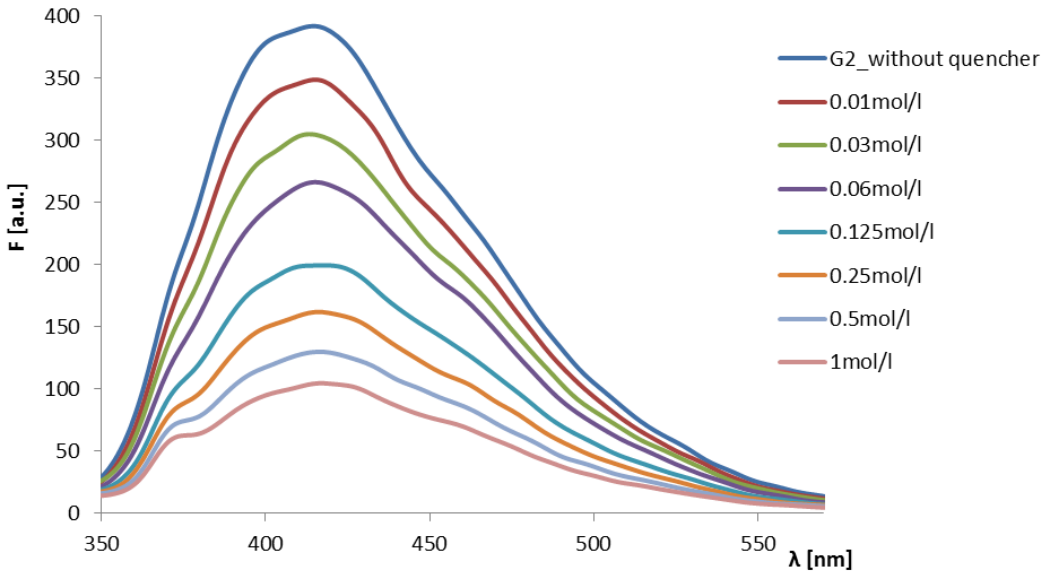

Fig. S7. Fluorescence quenching of PAMAM dendrimer G2 (c=1mmol/L) by KI.

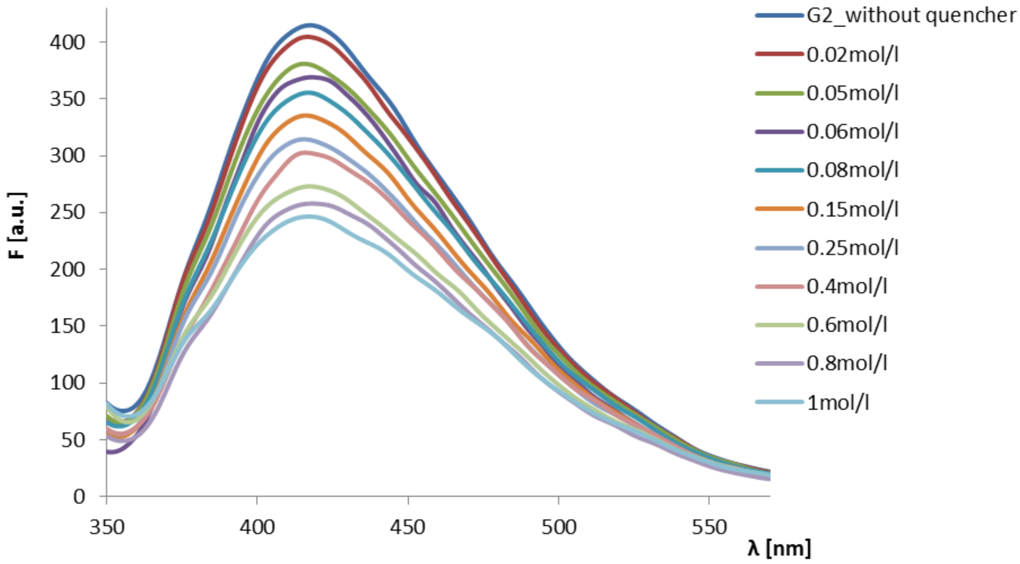

Fig. S8. Fluorescence quenching of PAMAM dendrimer G2 (c=1mmol/L) by acrylamide.
